# Supplementary material for: Drug repurposing for aging research using model organisms
Source: Aging Cell. 2017 Jun 16;16(5):1006–15. doi: 10.1111/acel.12626 (PMC5595691; doi:10.1111/acel.12626)
Supplement: Supplementary file 7 — Data S1 Zip‐Archive of all report cards. [file ACEL-16-1006-s007.zip › RC_CPT.pdf]

CPT

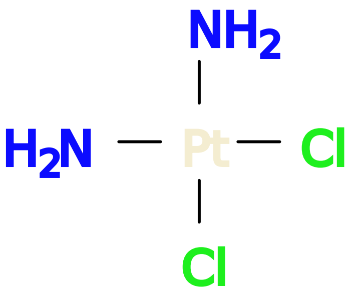

Database identifiers

ChEMBLCompound	CHEMBL1386
DrugBank	DB00515
CHEBI	27899
CHEBI	35852
eMolecules	10182026
eMolecules	14089862
eMolecules	498535
eMolecules	11340779
eMolecules	880843

Ranking

	Rank	Score
Drosophila	340/697	0.438
C. elegans	270/591	0.205

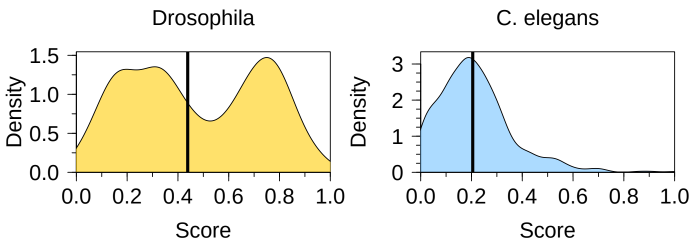

	Ageing implication	Domain conservation	Binding site conservation	Binding affinity	Bioavailability	Lipinski	Promiscuity	Purchasability	Drug approval	**Total**
Drosophila	1.0	0.922	0.553	0.575	(0.9)	0.0	-0.0	0.1	0.075	0.438
C. elegans	1.0	0.748	0.239	0.575	0.296	0.0	-0.0	0.1	0.075	0.205

Names

- **transplatin**
- Cisplatin
- Platinol
- trans-DDP
- trans-Diamminedichloroplatinum
- trans-Dichlorodiammine platinum
- trans-Platinum(II) ammonium chloride
- trans-Platinumdiammine dichloride

Roles

ChEBI entry 35852 has no roles

Status

Approved drug (according to ChEMBL)	No
Classification (according to DrugBank)	approved
Administration Route	Intravenous
Number of Rule of 5 violations	0
Binding affinity to original target in log units (RF-Score prediction)	5.3
Burns *C. elegans* bioavailability prediction	-0.5

Medical Information

Indicatation: For the treatment of metastatic testicular tumors, metastatic ovarian tumors and advanced bladder cancer.
Mechanism of action: Alkylating agents work by three different mechanisms: 1) attachment of alkyl groups to DNA bases, resulting in the DNA being fragmented by repair enzymes in their attempts to replace the alkylated bases, preventing DNA synthesis and RNA transcription from the affected DNA, 2) DNA damage via the formation of cross-links (bonds between atoms in the DNA) which prevents DNA from being separated for synthesis or transcription, and 3) the induction of mispairing of the nucleotides leading to mutations.
Retail: APP Pharmaceuticals, APPD, Baxter International Inc., Bedford Labs, Ben Venue Laboratories Inc., Bristol-Myers Squibb Co. and 5 more suppliers
Cisplatin 1 mg/ml vial - 0.41 USD / ml

(Information from DrugBank)

Compound Target Characteristics

Superoxide dismutase [Cu-Zn]

Best gene implication in ageing for this target family came from gene P61851 annotated in UniProt release 2014_02. Annotation GO 8340 (determination of adult lifespan) was Inferred from Mutant Phenotype

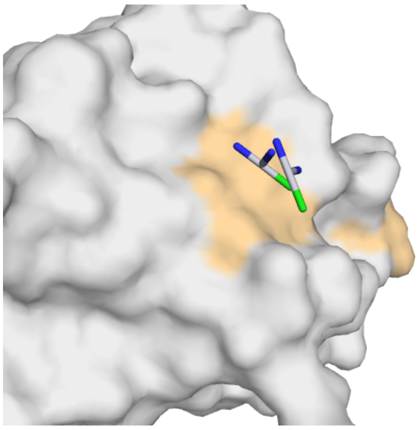

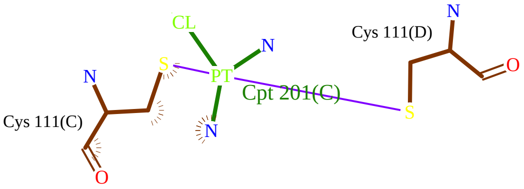

protein	amino acids contacts (binding site)			
PDB:3re0:chainA:P00441	E	L	C	I
PDB:3re0:chainC:P00441	E	L	C	I
tr:H7BYH4:H7BYH4_HUMAN	E	L	C	I
sp:P00441:SODC_HUMAN	E	L	C	I
sp:P07632:SODC_RAT	A	L	S	I
tr:Q6LDS4:Q6LDS4_RAT	A	L	S	I
sp:P08228:SODC_MOUSE	A	L	S	I
sp:P61851:SODC_DROME	S	L	S	I
tr:G5ECR5:G5ECR5_CAEEL	-	L	S	I
sp:P34461:SODE_CAEEL	-	L	S	I
sp:P00445:SODC_YEAST	S	L	S	V

	whole protein		domain-based		contact-based	
protein	ident	simil	ident	simil	ident	simil
PDB:3re0:chainA:P00441	1.0	1.0	1.0	1.0	1.0	1.0
PDB:3re0:chainC:P00441	1.0	1.0	1.0	1.0	1.0	1.0
tr:H7BYH4:H7BYH4_HUMAN	0.87	0.87	0.87	0.87	1.0	1.0
sp:P00441:SODC_HUMAN	1.0	1.0	1.0	1.0	1.0	1.0
sp:P07632:SODC_RAT	0.83	0.94	0.83	0.94	0.5	0.49
tr:Q6LDS4:Q6LDS4_RAT	0.82	0.93	0.82	0.93	0.5	0.49
sp:P08228:SODC_MOUSE	0.84	0.94	0.83	0.94	0.5	0.49
sp:P61851:SODC_DROME	0.61	0.84	0.6	0.85	0.5	0.55
tr:G5ECR5:G5ECR5_CAEEL	0.35	0.57	0.44	0.71	0.5	0.24
sp:P34461:SODE_CAEEL	0.35	0.57	0.44	0.71	0.5	0.24
sp:P00445:SODC_YEAST	0.54	0.83	0.55	0.84	0.25	0.55

Sod (FBgn0003462) associated phenotypes

calorie restriction conditional, chemical conditional, chemical sensitive, heat sensitive, increased cell number, locomotor behavior defective, long lived, male limited, oxidative stress response defective, partially, partially lethal - majority die, progressive, radiation sensitive, reduced, short lived

(Information from FlyBase)

Sod (UniProt:P61851) annotation

Function: Destroys radicals which are normally produced within the cells and which are toxic to biological systems.
Cofactor:Cu cationNote=Binds 1 copper ion per subunit.;
Cofactor: Zn(2+)Note=Binds 1 zinc ion per subunit.;
Subunit: Homodimer.
Subcellular location: Cytoplasm.

(Information from UniProt)

sod-4 (UniProt:G5ECR5) annotation

Function: Destroys radicals which are normally produced within the cells and which are toxic to biological systems. (RuleBase:RU000393).
Cofactor: Cu cation Evidence=(RuleBase:RU000393); Note=Binds 1 copper ion per subunit. (RuleBase:RU000393);
Cofactor: Zn(2+) Evidence=(RuleBase:RU000393); Note=Binds 1 zinc ion per subunit. (RuleBase:RU000393);

(Information from UniProt)

sod-4 (UniProt:P34461) annotation

Function: Destroys radicals which are normally produced within the cells and which are toxic to biological systems.
Cofactor:Cu cationNote=Binds 1 copper ion per subunit. ;
Cofactor: Zn(2+)Note=Binds 1 zinc ion per subunit. ;
Subcellular location: Isoform 1: Secreted, extracellular space.
Subcellular location: Isoform 2: Membrane ; Peripheral membrane protein

Tissue specificity: Isoform 2 is preferentially expressed in eggs.

(Information from UniProt)
